# Dendritic optical antennas: scattering properties and fluorescence enhancement

**DOI:** 10.1038/s41598-017-05108-3

**Published:** 2017-07-24

**Authors:** Ke Guo, Alessandro Antoncecchi, Xuezhi Zheng, Mai Sallam, Ezzeldin A. Soliman, Guy A. E. Vandenbosch, Victor. V. Moshchalkov, A. Femius Koenderink

**Affiliations:** 1 Center for Nanophotonics, AMOLF, Science Park 104, 1098 XG Amsterdam, The Netherlands; 2 Department of Electrical Engineering (ESAT-TELEMIC), KU Leuven, Kasteelpark Arenberg 10, BUS 2444, Leuven, B-3001 Belgium; 3 Department of Physics, American University in Cairo, AUC Avenue, P. O. Box 74, New Cairo, 11835 Egypt; 4 Laboratory of Solid State Physics and Magnetism, KU Leuven, Celestijnenlaan 200D, BUS 2444, Leuven, B-3001 Belgium

## Abstract

With the development of nanotechnologies, researchers have brought the concept of antenna to the optical regime for manipulation of nano-scaled light matter interactions. Most optical nanoantennas optimize optical function, but are not electrically connected. In order to realize functions that require electrical addressing, optical nanoantennas that are electrically continuous are desirable. In this article, we study the optical response of a type of electrically connected nanoantennas, which we propose to call “dendritic” antennas. While they are connected, they follow similar antenna hybridization trends to unconnected plasmon phased array antennas. The optical resonances supported by this type of nanoantennas are mapped both experimentally and theoretically to unravel their optical response. Photoluminescence measurements indicate a potential Purcell enhancement of more than a factor of 58.

## Introduction

Resonant optical nanoantennas^1^ enable efficient light matter interaction at nanometer scales, with large promise for a wide range of fields including light emission^2–7^, lasing^8–11^, sensing and nano-medicine^12–16^, photovoltaics^17–20^, and optical information processing^21–24^. Well-developed radio antenna theory has inspired the design of a variety of optical antennas such as dipole^25, 26^, bow-tie^11, 27^ and Yagi-Uda antennas^28^ in order to achieve different functionalities. In particular, narrow gaps between sharp structures are pursued for high local fields, while oligomers of plasmonic particles^29^ are designed to reach strong directivity and control over extinction and scattering spectra, on basis of plasmon hybridization and phased-array effects.

Contrary to many radio antennas, state-of-the-art optical antennas do not generally form a continuous electrical network, hampering electrical addressing of active matter that loads the antenna. Recently, Kern *et al*.^30^ for the first time created an electrically driven optical antenna in form of an optically connected bow-tie structure. Electrically connected antennas open the perspective of designing antennas that both provide optical enhancement and allow electric driving of active or nonlinear matter that loads the antenna. Similar perspectives could apply to linear and nonlinear metasurfaces to control the amplitude, phase, and spin/orbital angular momentum content of light^31, 32^, if they could be made in connected metallic form. Here we study the optical properties of antennas that are electrically connected and at the same time benefit from the plasmon hybridization effects known from hybridization of discrete particle plasmon resonances. In particular, we study so-called ‘dendritic’ antennas, that can be viewed as oligomers of nanorods that are electrically connected together to form a connected structure in a brickwork pattern. This design brings a dendritic RF antenna design that originally targeted RF communication and transduction, to the optics domain.

In this article, we study disconnected oligomers and the corresponding connected, dendritic antennas. Through mid-IR, IR and visible extinction spectroscopy, we map the optical resonances as function of the connectors, and as function of antenna ‘generation’, which quantifies how far the brickwork extends. We interpret the mode structure through two independent theoretical methods: the finite element method (FEM) allows to study driven antennas, while we use the volumetric method of moments (V-MoM) with group theoretical analysis to identify a classification scheme for the intrinsic eigenmodes. FEM simulation furthermore predicts large Purcell enhancements for emitters placed in a narrow gap that one could fabricate in the central antenna element. Finally, we present a preliminary, i.e., ensemble-averaged, photoluminescence (PL) brightness and decay rate enhancement study for overcoated high-efficiency, and low-efficiency organic dyes.

## Dendritic antenna design

**Figure 1:**
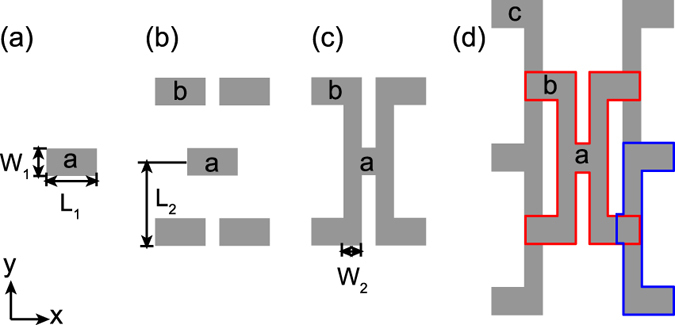
Schematics of (**a**) the single antenna element, (**b**) the disconnected pentamer, (**c**) the first and (**d**) second generation of dendritic antennas. The red and blue outlines in (**d**) indicate the first generation in (**b**) and an appended half of it respectively.

The basic design for dendritic optical antennas is illustrated in Fig. 1. Through an iterative growth rule one can build a hierarchy of antenna generations, where each generation spans a larger size. Starting with a single nanorod of length *L*_1_ (165 nm in our work) and width *W*_1_ (90 nm), we compose a pentamer of five identical, yet disconnected rods. For identification, we mark the central element a and the four added rods as b as shown in Fig. 1(a,b). The first generation of the dendritic optical antenna family is obtained by connecting the central element a to the side arms b with vertical connectors (geometrical parameters *L*_2_ = 140 nm *W*_2_ = 60 nm) as shown in Fig. 1(b,c). This design can be extended to generation *n* + 1 from generation *n* by adding a column of 2*n* + 1 arms (again of size *L*_1_, *W*_1_) to both the left and right, and again connecting those with vertical connectors. In principle an electrically connected network can be made in this way. This design is somewhat similar to fractal antennas^33, 34^. However, while fractals are generated by subdividing or filling a given area with ever smaller features, in our design the typical element size remains the same, but the full antenna size grows linearly with generation. One could envision that for sufficiently large generation number, one obtains a macroscopically sized connected electric network, with the original element *a* as a unique single-point constriction. In this work we study the first and second generation dendritic antenna in comparison with their unconnected counterparts, to elucidate the mode structure.

## Extinction

**Figure 2:**
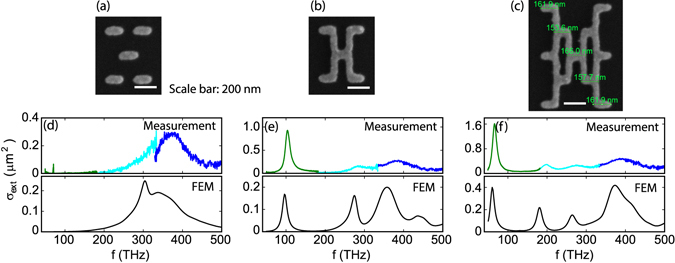
SEM images and comparison of measured (colored lines) and simulated (black lines) extinction cross sections of (**a**,**d**) the pentamer, (**b**,**e**) the first and (**c**,**f**) the second generation of dendritic antennas. The extinction cross section is measured with three detectors for different frequency ranges. Green: Bruker HYPERION Series FTIR; cyan: home-made FTIR; blue: Ocean optics fiber-coupled grating spectrometer.

Figure 2(a–c) show scanning electron microscope (SEM) images of an Au pentamer, and a first generation and second generation of dendritic antenna, all made by electron beam lithography. Alongside, Fig. 2(d–f) show the extinction cross sections per antenna for *x*-polarized driving (polarized along the rods) measured from three setups that we required to span a frequency range from 50 to 500 THz (see Methods). The measurement results from different setups agree well at their overlapping frequencies except for the pentamer case near 320 THz, where the FTIR operates at the﻿ lim﻿it﻿ ofits sensitivity range. The pronounced peaks in extinction cross sections indicate the different *x*-polarized optical modes supported by the antennas. As expected, the pentameter composed of unconnected rods only shows a response near 350 THz, i.e., near the single-rod dipole resonance. At similar frequencies two modes are found for the first and the second generations of the dendritic antennas. The first generation, however, also presents one new mode at a dramatically lower frequency around 100 THz, while the second generation offers two new modes around 180 THz and 60 THz respectively. In addition to the antennas studied here, we have studied the extinction of many similar antennas, i.e., antennas made by the same geometrical rule, but different geometrical parameters (arm length, connector length), and also made of Ag instead of Au. We generally find the same spectral progression, however with an overall redshift of features at larger geometrical parameters, as expected from size-scaling arguments.

To confirm the presence of distinct resonances in extinction, we conducted FEM simulations (COMSOL Multiphysics) on single antennas, assuming normally incident plane wave illumination with *x*-polarization. We calculate extinction as the sum of scattering and absorption in a total-field scattered-field calculation, including the glass substrate, and using optical constants for Au from Etchegoin ref. 35. The simulated extinction cross sections are shown in Fig. 2(d–f), each plotted below the corresponding measurement. Calculations in general accurately predict the resonances observed in the extinction measurements. Small shifts and disagreement in amplitude can be attributed to the difference between the simulated geometry and the actual sample geometry.

## Finite element mode analysis

Examining the calculated extinction in itself does not explain the underlying mode structure. Rather, one must inspect simulated near-fields and current densities. In this section, we discuss the mode assignments for the pentamer and the dendritic antennas one by one. We label the resonant modes obtained in the simulation as *n* − *m*, with *n* indicating the antenna generation (0 for pentamer), and *m* the mode number. For each mode we plot electric field and current distributions in a 2D cross section at select frequencies, and the volume-averaged induced current in *x*-direction.

**Figure 3:**
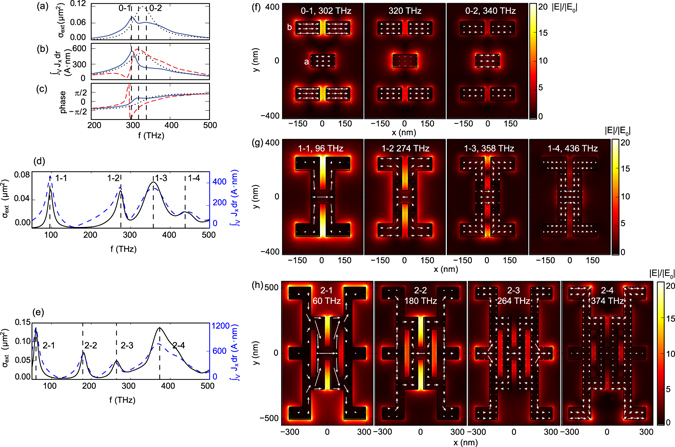
(**a**) Simulated extinction cross section of the pentamer (blue line) compared with that of a single rod multiplied by 5 (black dots). (**b**) Simulated amplitude and (**c**) phase of net current in the *x*-direction in a (blue line) and b (red dashed line) compared with the current in a single rod (black dots). (**d**,**e**) Simulated extinction coefficient (black line) and amplitude of net current in the *x*-direction (blue dashed line) of (**d**) the first and (**e**) the second generations of dendritic antennas. (**f**–**h**) Simulated maps of electrical field and current distribution in a cross section of (**f**) the pentamer, (**g**) the first and (**h**) the second generations of the dendritic antennas at resonant frequencies specified by the black dashed lines in (**a**–**e**).

Figure 3(a–c) and (f) show results for the pentamer which displays physics very similar to the well known plasmon heptamer^29, 36–39^. The pentamer has all its hybridized modes close to the single-rod resonance, i.e., near 300 THz, with a distinct Fano feature in extinction cross section that can be attributed a superradiant mode (all dipole moments aligned), and a subradiant mode (central arm has dipole moment against the outer arms), as shown in Fig. 3(a–c). The field and current plots (Fig. 3(f)) evidence that the response is dominated by the outer arms for f = 302 THz (peak labelled as 0–1 in Fig. 3(a–c)), with the central arm out of phase with the outer arms. At f = 340 THz (extinction peak labelled 0–2 in Fig. 3(a–c)), all arms respond in phase. At the intermediate frequency f = 320 THz, the central is a quarter cycle out of phase. While the Fano-feature in exctinction calculations is not as clear as for the heptamer^39^ due to imperfect tuning of the modes, these are the characteristic features of a plasmonic Fano resonance. The Fano feature, which does not constitute a main claim of this work, is not easily verified in our experiment due to the transition between two detector windows.

We can now understand the physics of the connected first generation antenna based on the pentamer modes. Figure 3(d) shows the simulated extinction cross section and net current in the x direction of the first generation of dendritic antenna. The lowest energy mode, Mode 1-1 has large current flowing from left to right through the entire antenna, without any node as shown in Fig. 3(g). Essentially, this mode can be seen as the fundamental *λ*/2 mode of a long rod, which one can envision by unfolding the three arms plus the two vertical connectors. This viewpoint is similar to the viewpoint on modes of folded metal structures first put forward by Rockstuhl *et al*. for split rings^40^. Mode 1–2 and Mode 1–3 directly derive from the subradiant and superradiant modes of the pentamer, both without current flowing in the connecting arms. The subradiant mode (central rod polarized against the outer ones) is redshifted while the superradiant mode is blue shifted. At even higher frequency a fourth mode appears in which the vertical connecting rods do participate (Mode 1–4).

Figure 3(e) and (h) shows how these results generalize to the second generation. The lowest order mode, Mode 2–1 is even further redshifted to 60 THz, and again shows currents flowing across the entire structure with no node. The longer wavelength is consistent with its larger size^40^. Modes 2–2 and Mode 2–3 (at 180 THz and 264 THz) can be considered as arising from Mode 1–2 (current in central arm opposed to its 4 neighbors), hybridized in turn either in phase or out of phase with the bright mode of the six outer arms. Finally, Mode 2–4 at 374 THz has currents in the same direction in all the arms with nodes in the connectors, and is therefore similar to the superradiant Mode 0–2 of the pentamer, and the superradiant Mode 1–3 of the first generation. The shoulder in the extinction spectrum above 400 THz indicates further higher order modes similar to mode 1–4, in which also the connecting rods carry current.

To summarize, we hypothesize that the following mode structure is responsible for the optical properties. First, the dendritic antennas have a fundamental *λ*/2 resonance that extends across the entire structure and hence redshifts with increasing generation number. Second, near 370 THz, the dendritic antennas have a mode in which all arms are in phase and the connecting rods are largely irrelevant, that coincides in character and in frequency with the superradiant mode of the pentamer. At frequencies in between the superradiant mode and the fundamental mode, various modes occur as hybrids of the pentamer subradiant mode with the outer arms.

## V-MoM with group theory analysis for classification of eigenmodes

The FEM simulation revealed resonant responses of dendritic antennas upon external driving by a plane wave. In any driven problem, one relies on a non-zero projection of the drive on the modes of the system to identify modes. In this section, we extend the mode assignment discussion in the previous section by combining an excitation independent eigenmode analysis with a group theory approach.

As this would be difficult to implement in COMSOL, we formulate the light interaction with a nano-scatterer in the framework of electric field - volume integral equations. (EF-VIEs)^41–45^ For the sake of conciseness, we summarize the electric field volume integral equation in an operator form (see the detailed forms in the supporting information),

1 $${\boldsymbol{Z}}({\boldsymbol{r}},{\boldsymbol{r}}{\boldsymbol{^{\prime} }};\omega )\cdot {\boldsymbol{J}}({\boldsymbol{r}}{\boldsymbol{^{\prime} }},\omega )={{\boldsymbol{E}}}_{{\boldsymbol{inc}}}({\boldsymbol{r}},\omega \mathrm{).}$$

In Eq. (1) ***J***(***r*** *′*, *ω*) represents the full solution, i.e. the induced current (and charge) flowing in a nanostructure due to an incident field ***E***_***inc***_, while the impedance operator ***Z***(***r***, ***r***′; *ω*) incorporates the Green’s function that describes how any part of the nanostructure (source point ***r***′) electromagnetically interacts with another part (at point ***r***). At any frequency, Eq. (1) can be analyzed as an eigenvalue problem^46^

2 $${\boldsymbol{Z}}({\boldsymbol{r}},{\boldsymbol{r}}{\boldsymbol{^{\prime} }};\omega )\cdot {{\boldsymbol{J}}}_{n}({\boldsymbol{r}}{\boldsymbol{^{\prime} }},\omega )={\lambda }_{n}(\omega ){{\boldsymbol{J}}}_{n}({\boldsymbol{r}}{\boldsymbol{^{\prime} }},\omega \mathrm{).}$$

In Eq. 2, ***J***(***r*** *′*, *ω*) is a complex spatial distribution which is independent of the incident field distribution. The corresponding eigenvalue *λ*_*n*_(*ω*) can be viewed as a generalized (inverse) polarizability. For any given incident field, the coupling coefficients *c*_*n*_ for coupling to each eigenvector are given by

3 $${c}_{n}(\omega )=\frac{{\int }_{V}{{\boldsymbol{J}}}_{n}({\boldsymbol{r}}{\boldsymbol{^{\prime} }},\omega )\cdot {{\boldsymbol{E}}}_{{\rm{inc}}}({\boldsymbol{r}}{\boldsymbol{^{\prime} }},\omega ){\rm{d}}{\boldsymbol{r}}{\boldsymbol{^{\prime} }}}{{\lambda }_{n}(\omega )},$$

where the integration runs over the antenna volume. By combining the contributions from all the eigenvectors, we are able to recover the full solution,

4 $${\boldsymbol{J}}({\boldsymbol{r}}{\boldsymbol{^{\prime} }},\omega )=\sum {c}_{n}(\omega ){{\boldsymbol{J}}}_{n}({\boldsymbol{r}}{\boldsymbol{^{\prime} }},\omega \mathrm{).}$$

Conveniently, in our implementation we can trace eigenvectors and their coupling coefficients continuously as function of frequency. When the magnitude of an eigenvalue becomes minimal, this implies a resonance in the induced current.

A distinct advantage of this formulation as an eigen-problem is that the finite-dimensional approximation to the impedance operator Z that appears in numerical analysis is amenable to symmetry analysis using group theory^47^. Our experimental system, i.e., a single structure on an air-glass interface, under normal incidence in *x*-polarization, implies *C*_2*v*_ symmetry, an Abelian group of order 4 with four irreducible representations. Combining the group’s irreducible representations and its transformation operators for vector fields, we can construct a set of projection operators^46, 48, 49^ ${{P}}_{j}$ that allow to block-diagonalize the impedance matrix as

5 $$Z=\underset{j=1}{\overset{4}{\oplus }}{Z}_{j},\quad {\rm{with}}\,{Z}_{j}={{P}}_{j}Z.$$

**Figure 4:**
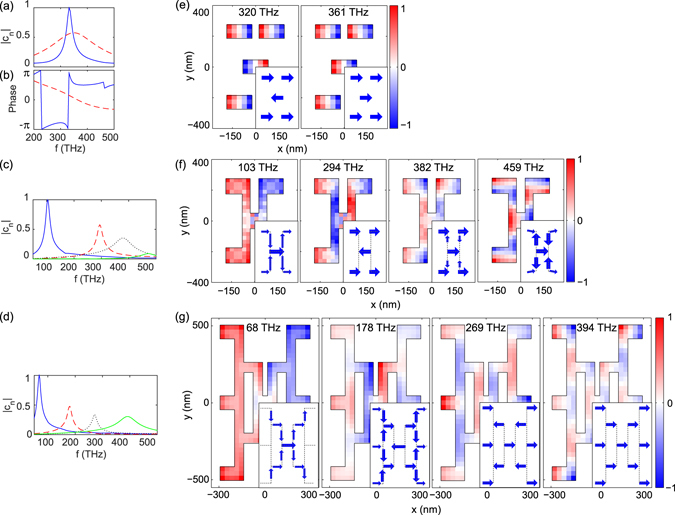
(**a**) Amplitude (normalized to the maximum) and (**b**) phase of the coupling coefficients of the the superradiant (blue solid lines) and subradiant (red dashed lines) modes supported by the pentamer. (**c**–**d**) Amplitude (normalized to the maximum) of the coupling coefficients of eigenmodes of (**c**) the first and (**d**) the second generations of the dendritic antennas. Normalized instantaneous charge distribution of eigenmodes obtained from V-MoM within the Γ_4_ representation for (**e**) the pentamer, (**f**) the first and (**g**) the second generations of the dendritic antennas. Insets illustrate the directions of instantaneous net current.

Instead of decomposing the eigenvalues of the original impedance operator as in Eq. (2), we can apply the eigenvalue decomposition to the projected matrices *Z*_*j*_, homing in on the eigenvectors and eigenvalues that belong within a certain irreducible representation. The excitation used in this work projects only on the irreducible representation Γ_4_, and accordingly only excites resonances in this representation. We note that oblique incidence, or incidence with different polarization would allow to excite also modes in several other irreducible representations. The strength of our experiment and the use of group theory is that by selecting the excitation condition according to symmetry, we focus on just a select set of modes. For the pentamer, out of a total of five modes, two fall within Γ_4_, corresponding to the subradiant and superradiant modes underlying Fano resonance as shown in Fig. 4(a,b) and (e). The modes within an irreducible representation are not necessarily orthogonal in an inner product sense^39, 46^ and can thus interfere to generate a Fano-line in extinction.

For the first and second generation of dendritic antennas, eigenmodes with similar frequencies and current distributions to the modes found by FEM simulation are obtained by the combination of group theory and V-MoM. For the first generation, we found 14 modes between 50 and 500 THz, of which five fall within Γ_4_. Figure 4(c) and (f) show the coupling coefficients and charge distributions for the first four resonances (with the lowest frequency, f = 103, 294, 382, and 459 THz). These results confirm that the fundamental mode is essentially a *λ*/2 resonance spanning the entire structure, that the mode at 294 THz is a (redshifted) subradiant mode, and that the resonance at 382 THz is essentially the original superradiant mode of the pentamer. The mode at 459 THz has significant y− oriented currents.

For the second generation we identified in total 36 modes between 50 and 500 THz of which most are not coupled to normal-incidence plane waves. Figure 4(d) and (g) show the first four resonances in Γ_4_ with a resonant coupling coefficient between 50 and 500 THz, again showing that the qualitative identification that we derived from the FEM simulations is correct and complete.

## Analysis of Purcell enhancement

Having identified the mode structure of dendritic antennas, we ask whether they are interesting for spontaneous emission control. As a starting point, the rationale is that if one would couple a single emitter to the central nanorod, the pentamer superradiant mode would aid in obtaining bright emission. Further, if one would replace the central nanorod of the pentamer by a nanorod with a narrow gap in the center, one could at the same time benefit from the high LDOS in the narrow gap^1^, and the superradiant dipole physics of the pentamer. Since the dendritic antennas support essentially the same superradiant modes, it is interesting to ask if dendritic antennas retain or improve the dipole array antenna physics. If so, an advantage of dendritic antennas would be electrical addressability of the gap where the emitter sits.

Optical antennas can modify various photoluminescence observables through different mechanisms that trade off depending on emitter efficiency. They can (1) enhance the absorption of pump light, (2) modify radiation patterns, (3) accelerate emission, and (4) introduce additional quenching due to absorption by the metal. The joint effect of (3) and (4) depends on intrinsic quantum yield (QY_0_) of the fluorophores chosen. Intrinsically high QY_0_ allows to accelerate decay by the Purcell effect, with a drawback in efficiency due to the quenching. In contrast, an inefficient emitter (QY_0_ << 1) will gain in efficiency when Purcell enhanced rates out-compete intrinsic nonradiative rates, even if the total decay rate is only modestly affected. Expressed mathematically, we have the following expectations for the rate enhancement^50^

6 $$\frac{\gamma }{{\gamma }_{0}}\approx \{\begin{array}{cc}{\rho }_{tot}, & {\rm{when}}\,{{\rm{QY}}}_{0}\approx 1\\ \mathrm{1,} & {\rm{when}}\,{{\rm{QY}}}_{0}\approx \mathrm{0,}\end{array}$$

resp. the change in emission efficiency

7 $$\frac{{\rm{QY}}}{{{\rm{QY}}}_{0}}\approx \{\begin{array}{cc}{\rho }_{r}/{\rho }_{tot}, & {\rm{when}}\,{{\rm{QY}}}_{0}\approx 1\\ {\rho }_{r}, & {\rm{when}}\,{{\rm{QY}}}_{0}\approx \mathrm{0,}\end{array}$$

where *ρ*_*tot*_ indicates the enhancement of total emission rate, i.e., the local density of states of which the radiative part is *ρ*_*r*_.

**Figure 5:**
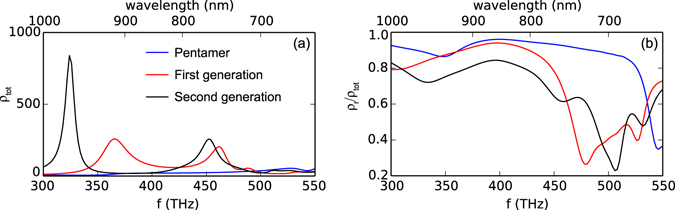
(**a**) Total decay rate enhancement *ρ*_*tot*_ from the Ag pentamer, first and second generation of dendritic antennas and (**b**) *ρ*_*r*_/*ρ*_*tot*_, i.e., maximum achievable antenna quantum efficiency, as simulated by FEM.

We perform FEM simulations assuming a single dipole source oriented in the x direction in the center of the antennas, in a 30 nm gap. For fluorescence measurements as reported below, one typically requires covering the antennas by a thin polymer layer that is doped with fluorophores. To compensate the red-shifts of antenna resonances due to polymer coverage, we use Ag instead of Au antennas, to maintain the resonances at the visible and near-infrared range to be comparable with most fluorophores. Extinction calculations verify that swapping the material from Au to Ag, overcoating with polymer, and creating a narrow gap, leaves the character of the modes unchanged modulo a frequency shift. Figure 5 shows the calculated *ρ*_*tot*_ and *ρ*_*r*_/*ρ*_*tot*_ on Ag antennas. Referring to Eqs 6 and 7, the results demonstrate that while the pentamer gives a modest enhancement (below 100) in emission rate over the simulated frequency range, the dendritic antennas provide much larger enhancements (more than 200) accompanied by a drop of efficiency by 20% to 80%.

## Fluorescence experiment

Instead of an ideal experiment using single fluorophores, we have performed a preliminary, ensemble-level experiment on Ag pentamers, first- and second- generation dendritic antennas with a 30 nm gap in the center. Conversely, the brightness of emission for intrinsically low-efficiency dye reports on a radiative local density of states. In practice, in such a measurement one would measure PL enhancement (PLE) from the antennas which consists of three contributions:

8 $${\rm{PLE}}=\frac{{\rm{PL}}}{{{\rm{PL}}}_{0}}=\frac{{\rm{A}}}{{{\rm{A}}}_{0}}\cdot \frac{{\rm{D}}}{{{\rm{D}}}_{0}}\cdot \frac{{\rm{QY}}}{{{\rm{QY}}}_{0}},$$

where A and A_0_ denote the absorption of pump light with and without antennas, D and *D*_0_ denote collection efficiencies per radiated photon and QY is the QY modified due to Purcell effect and quenching. Measurement of PLE on a single sample cannot be easily separated into its constituent effects, but measurements in two regimes of QY aids unraveling the physics determining PLE. To do this, We make use of a dye system that can operate with both low and reasonably high efficiencies, depending on the thermal treatment on the sample.

**Figure 6:**
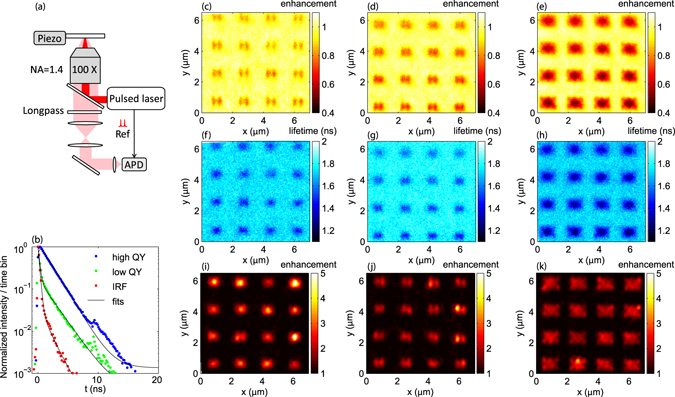
(**a**) A schematic of the confocal time-correlated single-photon counting setup for PL measurements. (**b**) Measured decay curves of the baked and unbaked dye layers, IRF of the laser and the fits to them. (**c**–**e**) PL enhancement and (**f**–**h**) lifetime measured from unbaked dye layers on (**c**,**f**) the pentamer, (**d**,**g**) first and (**e**,**h**) second generation of dendritic antennas. (**i**–**k**) PL enhancement measured from baked dye layers on (**i**) the pentamer, (**j**) first and (**k**) second generation of dendritic antennas.

The dye molecules we use are Rh 800 molecules (emitting at 710 nm) doped in polymer (polystyrene) with a weight concentration of 10%, and spincoated in a 45 nm layer. After spin-coating, the sample is either only exposed to ambient conditions, or baked at 150 °C for 30 min prior to optical measurements. Baking promotes solvent evaporation and polymer crosslinking, meaning a physical and chemical change in the environment of the dye molecules. Figure 6(b) shows the measured fluorescence decay with and without baking measured with a confocal microscope illustrated in Fig. 6(a) (details reported in the Method section). The unbaked dye layer exhibits a single exponential decay, which can be fitted with the convolution of the instrument response function (IRF) and a 1.7 ns decay. As reported in ref. 51 Rh 800 molecules have a QY of 0.25 and a lifetime of 1.93 ns in absolute ethanol, meaning a radiative decay rate of 0.13 ns^−1^. Accounting for the refractive index of polystyrene, one would expect a radiative decay rate of 0.15 ns^−1^ in the unbaked dye layer. Hence the 1.7 ns decay time is consistent with an essentially unchanged quantum efficiency. The baked dye layer exhibits a double exponential decay. The fast decay channel almost overlaps with the IRF, indicating a much shorter lifetime than the IRF lifetime which therefore cannot be accurately fitted. The slow decay channel follows the decay curve of the unbaked dye layer. Therefore, we understand the baked dye layer as containing two molecular species: a fraction unaffected by the thermal treatment, and a fraction that has a significantly lower QY. From a double exponential fit we derive the ratio of the numbers of photons from the low QY dye molecules and the high QY dye molecules to be *α*_1_:*α*_*h*_ ≈ 0.54:1.

We perform confocal imaging of PL intensity and lifetime at the antenna array as shown in Fig. 6. For the unbaked dye layer (Fig. 6(c–h)), the PL intensity hardly changes in the center of the pentamer and is reduced in the center of the first generation and second generation by 30% and 60% respectively. This reduction is the product of pump field enhancement/reduction, directivity and quenching induced by the antennas. The lifetime is reduced by about 20% (pentamer and first generation) to 50% (second generation) as shown in Fig. 6(f–h), which respectively correspond to *ρ*_*tot*_ ≈  2 and 3 compared to the total density of states without antennas, if one accounts for QY = 0.25.

In contrast with the unbaked dye layer, the emission of the baked dye layer is strongly enhanced near the center of the antennas as shown in Fig. 6(i–k). This behavior is consistent with the notion that for poor efficiency emitters only, Purcell enhancement can express itself as a brightness enhancement. Considering the different contribution of the low and high QY dye molecules, the PLE of the baked dye layer can be expressed as:

$${{\rm{PLE}}}^{{\rm{baked}}}=\frac{{\rm{A}}}{{{\rm{A}}}_{0}}\cdot \frac{{\rm{D}}}{{{\rm{D}}}_{0}}\cdot \frac{1}{{\alpha }_{l}+{\alpha }_{h}}\cdot ({\alpha }_{l}{\rho }_{r}+\frac{{\alpha }_{h}{\rho }_{r}}{1+({\rho }_{tot}-\mathrm{1)}{{\rm{QY}}}_{0}^{h}}),$$

where ${{\rm{QY}}}_{0}^{h}\approx 0.25$ is the QY of the high QY dye molecules. Comparing the PLE of the baked and unbaked dye layers enables separation of the total decay rate enhancement from the influence of the pump enhancement and the modification of radiation pattern with:

9 $$\frac{{{\rm{PLE}}}^{{\rm{baked}}}}{{{\rm{PLE}}}^{{\rm{unbaked}}}}\approx \frac{{\alpha }_{l}\mathrm{(1}+({\rho }_{tot}-\mathrm{1)}{{\rm{QY}}}_{0}^{h})+{\alpha }_{h}}{{\alpha }_{l}+{\alpha }_{h}},$$

where the effect of directivity and pump field enhancement drop out. From Fig. 6, we estimate PLE^baked^/PLE^unbaked^ to be 3.5, 2.8 and 6.0 respectively for the pentamer, and the first and second generation dendritic antenna. Accordingly, the measured change in local density of states *ρ*_*tot*_ amounts to 30, 22 and 58 for the three antennas.

We note that the measurements have been conducted on a 45 nm layer with a confocal resolution of about 300 nm. Due to averaging, the data provides a lower bound to the local density of states at the antenna center. In the low- and high-QY measurement scenario, the weighting is quite different. For the measurement on the unbaked sample (efficient emitters), a dominant effect is that emitters with the most strongly enhanced rate are underrepresented, as their efficiency drops due to quenching. Consequently, the *ρ*_*tot*_ obtained from the lifetime is much lower than the actual *ρ*_*tot*_ in the center of the antennas. Conversely, for the low-QY molecules in the baked sample, the emission efficiency for emitters at the antenna center is enhanced. Therefore, the *ρ*_*tot*_ obtained from the PLE gives a much higher estimate for the LDOS change. The results from both the lifetime and PLE measurement show that all the investigated antennas are able to enhance the emission rate of a low efficiency dye near 710 nm. The second generation of the dendritic antenna has a much higher enhancement than the pentamer and the first generation, as in the simulation in Fig. 5.

## Conclusion

We have fabricated a new type of electrically connected antennas devised according to a generic growth rule that can provide space-filling dendritic networks. These antennas are potentially suitable for optically and electrically functional devices. Using extinction spectroscopy, we have measured the optical response of the pentamer, the first and second generation of the dendritic antennas in mid-IR, IR and visible. The measured optical resonances are identified using two independent theoretical methods (FEM and V-MoM). With FEM simulation, we have furthermore found the potential of using these modes to enhance the emission efficiency of emitters placed in a narrow gap in the center of the antennas. This enhancement is confirmed by preliminary measurements on the PL intensity and lifetime of thin dye-doped polymer layers on the antennas.

## Methods

### Fabrication protocol, extinction setups

Samples are fabricated on silica substrates using electron beam lithography (20 kV, ZEP-resist), evaporation of 30 nm of Ag or Au, and subsequent liftoff. To obtain reasonable signal strength in ensemble transmission measurements, the antennas are arranged in dilute square arrays with a pitch of 1.75 μm. We measured transmittances over a huge spectral range of *f* = 50–500 THz (6 μm to 0.6 μm in wavelength) with polarization parallel to the antenna arms. This large range turned out to be required due to the inverse scaling of antenna fundamental frequency, and the length over which charge separation occurs^40, 52^. The large spectral range required three different setups. An Ocean Optics fiber-coupled grating spectrometer was used for the visible to near-infrared spectrum (*f* = 320–500 THz). In this setup, we use a fiber-coupled halogen lamp, and measured on sample areas of about 50 μm diameter. For lower frequency, we use Fourier transform infrared (FTIR) spectroscopy. To cover the range from 50 to 150 and 150 to 330 THz, we used NA = 0.4 reflective optics and a HgCdTe detector for the mid-IR, and transmissive optics with an InGaAs diode for the intermediate frequency range. We convert transmission *T* into effective extinction cross sections per antenna through *σ*_ext_(*f*) = (1 − *T*) · *A*_cell_, with *A*_cell_ = 3.1 μm^2^ the unit cell area of our arrays.

### Confocal PL setup

We measure the PL intensity and lifetime with a confocal microscope, the schematic of which is shown in Fig. 6(a). A pulsed diode laser with a wavelength of 650 nm and a repetition rate of 10 MHz (Picoquant, ps pulses) is used to excite the dye molecules. The emission to the substrate side is collected by an objective with NA = 1.4 and detected by an avalanche photodiode (APD) after passing a longpass filter at 680 nm. The APD (IdQuantique id100-20ULN) measures the PL intensity and lifetime based on time-correlated single photon counting (Becker & Hickl, DPC230).

### Fluorescence analysis

Mathematically, suppose that we start with an emitter with nonradiative decay rate *γ*_*non*_ and radiative decay rate *γ*_*r*_ so that QY_0_ = *γ*_*r*_/(*γ*_*non*_ + *γ*_*r*_). If we place this emitter in an environment which provides a local density of states change *ρ*_*tot*_ of which the radiative part is *ρ*_*r*_, the change of rate of emission can be written as

10 $$\begin{array}{rcl}\frac{\gamma }{{\gamma }_{0}} & = & \frac{{\gamma }_{r}{\rho }_{tot}+{\gamma }_{non}}{{\gamma }_{r}+{\gamma }_{non}}\\  & = & 1+({\rho }_{tot}-\mathrm{1)}{{\rm{QY}}}_{0}\\  & \approx  & \{\begin{array}{cc}{\rho }_{tot}, & {\rm{when}}\,{{\rm{QY}}}_{0}\approx 1\\ \mathrm{1,} & {\rm{when}}\,{{\rm{QY}}}_{0}\approx \mathrm{0,}\end{array}\end{array}$$

while the change of QY due to the antennas can be expressed as

11 $$\begin{array}{rcl}\frac{{\rm{QY}}}{{{\rm{QY}}}_{0}} & = & \frac{{\rho }_{r}{\gamma }_{r}}{{\rho }_{tot}{\gamma }_{r}+{\gamma }_{non}}\cdot \frac{{\gamma }_{r}+{\gamma }_{non}}{{\gamma }_{r}}\\  & = & \frac{{\rho }_{r}}{1+({\rho }_{tot}-\mathrm{1)}{{\rm{QY}}}_{0}}\\  & \approx  & \{\begin{array}{cc}{\rho }_{r}/{\rho }_{tot}, & {\rm{when}}\,{{\rm{QY}}}_{0}\approx 1\\ {\rho }_{r}, & {\rm{when}}\,{{\rm{QY}}}_{0}\approx 0.\end{array}\end{array}$$

Comparing the PLE for the baked and unbaked sample results in

12 $$\begin{array}{rcl}\frac{{{\rm{PLE}}}^{{\rm{baked}}}}{{{\rm{PLE}}}^{{\rm{unbaked}}}} & = & \frac{1}{{\alpha }_{l}+{\alpha }_{h}}\cdot ({\alpha }_{l}{\rho }_{r}+\frac{{\alpha }_{h}{\rho }_{r}}{1+({\rho }_{tot}-\mathrm{1)}{{\rm{QY}}}_{0}^{h}})\cdot \frac{1+({\rho }_{tot}-\mathrm{1)}{{\rm{QY}}}_{0}^{h}}{{\rho }_{r}}\\  & \approx  & \frac{{\alpha }_{l}\mathrm{(1}+({\rho }_{tot}-\mathrm{1)}{{\rm{QY}}}_{0}^{h})+{\alpha }_{h}}{{\alpha }_{l}+{\alpha }_{h}}\mathrm{.}\end{array}$$

where directivity and pump enhancement have dropped out.

## Electronic supplementary material

Supplementary information
