# Supplementary material for: Dendritic optical antennas: scattering properties and fluorescence enhancement
Source: Sci Rep. 2017 Jul 24;7:6223. doi: 10.1038/s41598-017-05108-3 (PMC5524762; doi:10.1038/s41598-017-05108-3)
Supplement: Supplementary file 1 — Supplementary information [file 41598_2017_5108_MOESM1_ESM.pdf]

Supplementary information

Dendritic optical antennas: scattering properties and fluorescence enhancement

Ke Guo[1], Alessandro Antoncecchi[1], Xuezhi Zheng[2], Mai Sallam[2,3], Ezzeldin A. Soliman[3], Guy A. E. Vandenbosch[2], Victor. V. Moshchalkov[4], and A. Femius Koenderink[1]

[1]Center for Nanophotonics, AMOLF, Science Park 104, 1098 XG Amsterdam, The Netherlands

[2]Department of Electrical Engineering (ESAT-TELEMIC), KU Leuven, Kasteelpark Arenberg 10, BUS 2444, Leuven, B-3001, Belgium

[3]Department of Physics, American University in Cairo, AUC Avenue, P. O. Box 74, New Cairo 11835, Egypt

[4]Laboratory of Solid State Physics and Magnetism, KU Leuven, Celestijnenlaan 200D, BUS 2444, Leuven, B-3001, Belgium

S1. Formulating the interaction of light with a nanoscatterer in the framework of a Volume Integral Equation (VIE)

In the following, we discuss a Volume Integral Equation (VIE) formalism for light – nanostructure interactions. This material can also be found in our previous work [1]. It is reviewed here only for the sake of completeness. The physical process governing the interaction between light and a general scatterer can be described by the following two equations in the frequency domain,

$$\mathbf{E}_{\mathbf{tot}}(\mathbf{r}) = \mathbf{E}_{\mathbf{inc}}(\mathbf{r}) + \mathbf{E}_{\mathbf{scat}}(\mathbf{r}),\ \mathbf{E}_{\mathbf{scat}}(\mathbf{r}) = i\omega\mu_0\mu_1\int_V \overleftrightarrow{G}(\mathbf{r},\mathbf{r}')\cdot\mathbf{J}(\mathbf{r}')dv'. \quad \text{(S1)}$$

In Eqs. (S1), the first equation simply states that everywhere in space the total electric field is the sum of the *impressed* incident field and the scattered field. This scattered field is generated by the induced currents flowing in the source volume, which gives the gist of the second equation in Eqs. (S1). Here, μ_0 and μ_1 are the vacuum permeability and the relative permeability of the material filling the space where the scatterer is situated. $\bar{G}(\mathbf{r},\mathbf{r}')$ is the electric dyadic Green's function. Please note that in this article a $e^{-i\omega t}$ time convention is employed and the angular frequency ω has been systematically suppressed. Especially at the spatial position of the scatterer, the total field is linked with the induced current via,

$$\mathbf{E}_{\mathbf{tot}}(\mathbf{r}) = \frac{\mathbf{J}(\mathbf{r})}{-i\omega\varepsilon_0\left(\varepsilon_r(\omega)-1\right)},\ \mathbf{r}\in V. \quad \text{(S2)}$$

V in Eqs. (S1) and Eq. (S2) denotes the source volume. In Eq. (S2), ε_0 and $\varepsilon_r(\omega)$ represent the vacuum permittivity and the relative permittivity of the material that constitutes the scatterer. Combining the above equations, we have

$$\frac{\mathbf{J}(\mathbf{r})}{-i\omega\varepsilon_0\left(\varepsilon_r(\omega)-1\right)}-i\omega\mu_0\mu_1\int_V \overleftrightarrow{G}(\mathbf{r},\mathbf{r}')\cdot\mathbf{J}(\mathbf{r}')dv'=\mathbf{E}_{\mathbf{inc}}(\mathbf{r}),\ \mathbf{r}\in V. \tag{S3}$$

In Eq. (S3), since the incident electric field and the electric dyadic Green's function are assumed to be known in the first place, the induced current is the main target to solve and can be numerically evaluated by, e.g. a Method of Moments (MoM) algorithm. Writing compactly, we have the following operator formalism as in the main text,

$$Z(\mathbf{r},\mathbf{r}';\omega)\cdot\mathbf{J}(\mathbf{r}',\omega)=\mathbf{E}_{\mathbf{inc}}(\mathbf{r},\omega). \tag{S4}$$

In Eq. (S4), the impedance operator $\mathbf{Z}(\boldsymbol{r},\boldsymbol{r}';\omega)$ is

$$Z(\mathbf{r},\mathbf{r}';\omega)=\frac{1}{-i\omega\varepsilon_0\left(\varepsilon_r(\omega)-1\right)}\delta(\mathbf{r}-\mathbf{r}')-i\omega\mu_0\mu_1\int_V \overleftrightarrow{G}(\mathbf{r},\mathbf{r}')\cdot\ dv'. \tag{S5}$$

A Dirac delta is added in Eq. (S5) to emphasize the local approximation.

S3. Group theory considerations

Although the unconnected, 1st generation and 2nd generation Dendritic antennas have very different geometries, the symmetry operations underlying these geometries are unchanged. These symmetry operations (See Fig. S1(a-b)) are the same as the ones for a 2D rectangle: the identity operation E where no transformation is conducted, a rotation of π about the *z* axis, a mirroring operation m_y with respect to the *y* axis, and a mirroring operation m_x with respect to the *x* axis. These symmetry operations can be proved to form a group which is a C_{2v} group. The order of the group *h*, i.e. the number of elements in this group, is four.

(a) (b)

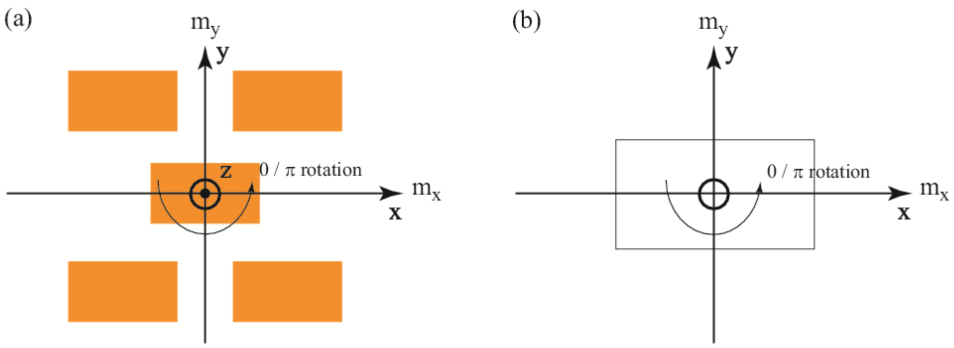

(c)

	Γ_1	Γ_2	Γ_3	Γ_4
**E**	+1	+1	+1	+1
**$C_2(z)$**	+1	+1	-1	-1
**m_y**	+1	-1	+1	-1
**m_x**	+1	-1	-1	+1

Fig.S1. Illustration of the symmetry operations for an unconnected structure. (a) and (b) demonstrate the symmetry operations for an unconnected structure and a rectangle. These symmetry operations form a group and the irreducible representations of the group are shown in Fig. 1(c).

Since symmetry operations are always applied based on coordinates, we should be able to find a corresponding set of matrices that “represent” these operations. Here, we especially focus on the matrices with the lowest dimensionalities, that is, the irreducible representations. Since the group under discussion is an Abelian group, we have four irreducible representations and they are shown in Fig. S1(c). Moreover, in contrast to these transformations operating on coordinates, we follow Wigner’s conventions [7-9] and define transformation operators which operate on functions,

$$P_R f(\mathbf{r}) = f(R^{-1}\mathbf{r}),\; P_R \mathbf{f}(\mathbf{r}) = R \cdot \mathbf{f}(R^{-1}\mathbf{r}). \tag{S6}$$

In Eq. (S6), this definition is illustrated for both scalar functions (such as charge, etc.) and vector functions (such as currents, electromagnetic fields, etc.). These transformation operators are commutative with the impedance operator defined in Eq. 3 of the main text (i.e., Eq. S4,S5)

Combining the group’s irreducible representations and its transformation operators, we can further construct a set of projection operators [7-9] for the group under discussion,

$$P_j = \frac{l_j}{h} \sum_R \Gamma_j(R)^* P_R. \tag{S7}$$

In Eq. (S7), a projection operator is characterized by the subscript j which marks an irreducible representation. Here, j may run from one to four. l_j is the dimensionality for an irreducible representation and since every irreducible representation has a dimensionality of one, l_j is equal to one. Then, the summation is carried out with respect to all the symmetry operations.

S3. On the commutative relation between an impedance operator and transformation operators

In this section, we prove the fact that the impedance operator defined in Eq. (S4) is indeed commutative with a transformation operator,

$$Z(\mathbf{r},\mathbf{r}';\omega)\cdot P_R\mathbf{J}(r')=P_R Z(\mathbf{r},\mathbf{r}';\omega)\cdot\mathbf{J}(r'). \tag{S8}$$

As we consider a specific frequency, in the following we will systematically suppress the frequency variable appearing in Eq. (S8). We assume that the targeted structure holds some symmetry operation *R.* In accordance with this symmetry operation we can define a transformation operator which works on functions, for example, the current in Eq. (S1-S4),

$$P_R\mathbf{J}(\mathbf{r}')=R\cdot\mathbf{J}\left(R^{-1}\mathbf{r}'\right). \tag{S9}$$

In this work the structure is put on top of a glass substrate occupying the lower half space. Consequently, (1) the symmetry operation and its corresponding transformation operator are actually confined to the *x-y* plane. For example, the symmetry operation can be represented by a matrix,

$$R=\begin{pmatrix} R_{xx} & R_{xy} & 0 \\ R_{yx} & R_{yy} & 0 \\ 0 & 0 & 1 \end{pmatrix}. \tag{S10}$$

Especially, here we focus on two types of elementary transformations in the *x-y* plane: rotations about the origin by an angle θ and reflections about a line which makes an angle θ with the *x* axis,

$$R_{rot}=\begin{pmatrix} \cos\theta & -\sin\theta & 0 \\ \sin\theta & \cos\theta & 0 \\ 0 & 0 & 1 \end{pmatrix},\ R_{refl}=\begin{pmatrix} \cos 2\theta & \sin 2\theta & 0 \\ \sin 2\theta & -\cos 2\theta & 0 \\ 0 & 0 & 1 \end{pmatrix}. \tag{S11}$$

In Eq. (S11), the matrices are orthogonal matrices with a determinant either +1 or -1. Other more complex transformations, for example, inversions, can be constructed by combining the above two elementary operations.

Moreover, due to the presence of the lower half space, we can split the dyadic Green's function in Eq. (S5) into two parts, that is, a direct wave part and a reflected wave part,

$$\overline{G}(\mathbf{r},\mathbf{r}')=\overline{G}_0(\mathbf{r},\mathbf{r}')+\overline{G}_r(\mathbf{r},\mathbf{r}'). \tag{S12}$$

For the direct field part, a closed form expression in the spatial domain is

$$\overline{G}_0(\mathbf{r},\mathbf{r}')=\left[I+\frac{\nabla\nabla}{k^2}\right]g(\mathbf{r},\mathbf{r}')=\left[I+\frac{\nabla\nabla}{k^2}\right]\frac{e^{ikr}}{r}. \tag{S13}$$

It can be seen from the last expression in Eq. (S13) that $g(\boldsymbol{r},\boldsymbol{r}')$ is only dependent on the distance between observation and source point and thus can be replaced by $g(|\boldsymbol{r}-\boldsymbol{r}'|)$. Correspondingly, we

apply the impedance operator that only regards the direct field interaction to an arbitrary current distribution that is operated on by a transformation operator P_R,

$$\int_V \bar{G}_0(\mathbf{r},\mathbf{r}')\cdot P_R\mathbf{J}(\mathbf{r}')dv' = \int_V \left[I+\frac{\nabla\nabla}{k^2}\right] g\left(\left|\mathbf{r}-\mathbf{r}'\right|\right)\cdot R\cdot\mathbf{J}\left(R^{-1}\mathbf{r}'\right)dv'. \tag{S14}$$

To tackle Eq. (S14), we perform a change of variables to the original source coordinate system, i.e. $\mathbf{x}' = R^{-1}\boldsymbol{r}'$,

$$\begin{aligned}\int_V \left[I+\frac{\nabla\nabla}{k^2}\right] g\left(\left|\mathbf{r}-R\mathbf{x}'\right|\right)\cdot R\cdot\mathbf{J}(\mathbf{x}')\left|\det(R)\right|d\tau' &= \int_V \left[I+\frac{\nabla\nabla}{k^2}\right] g\left(\left|\mathbf{r}-R\mathbf{x}'\right|\right)\cdot R\cdot\mathbf{J}(\mathbf{x}')d\tau' \\ &= \int_V \left[I+\frac{\nabla\nabla}{k^2}\right] g\left(\left|R^{-1}\mathbf{r}-\mathbf{x}'\right|\right)\cdot R\cdot\mathbf{J}(\mathbf{x}')d\tau'\end{aligned} \tag{S15}$$

In the above derivation, we use the following facts:

1) Since we have changed variables, a Jacobian must appear in combination with the infinitesimal element, that is, $dv' = |det(R)|\cdot d\tau'$. However, the symmetry operation *R* is represented by an orthogonal matrix. Since the absolute value of the determinant of an orthogonal matrix is 1, this term is dropped in the second expression.
2) The integral limits in Eq. (S15) are unchanged only because we assume that the structure is invariant under the symmetry operation *R*.
3) To reach the last expression, we notice that any orthogonal transformation does not affect the distance between two points.

Notice that the gradient operator in Eq. (S15) is taken with respect to the original observation coordinate system. In the transformed coordinate, the gradient operator reads,

$$\begin{aligned}\nabla\cdot\mathbf{f}\left(R^{-1}\mathbf{r}\right) &= \frac{\partial}{\partial x}f_x\left(R^{-1}\mathbf{r}\right)+\frac{\partial}{\partial y}f_y\left(R^{-1}\mathbf{r}\right)+\frac{\partial}{\partial z}f_z\left(R^{-1}\mathbf{r}\right) \\ &= \frac{\partial}{\partial X}f_x(\mathbf{x})\frac{\partial X}{\partial x}+\frac{\partial}{\partial Y}f_x(\mathbf{x})\frac{\partial Y}{\partial x}+\frac{\partial}{\partial Z}f_x(\mathbf{x})\frac{\partial Z}{\partial x} \\ &\quad+\frac{\partial}{\partial X}f_y(\mathbf{x})\frac{\partial X}{\partial y}+\frac{\partial}{\partial Y}f_y(\mathbf{x})\frac{\partial Y}{\partial y}+\frac{\partial}{\partial Z}f_y(\mathbf{x})\frac{\partial Z}{\partial y} \\ &\quad+\frac{\partial}{\partial X}f_z(\mathbf{x})\frac{\partial X}{\partial z}+\frac{\partial}{\partial Y}f_z(\mathbf{x})\frac{\partial Y}{\partial z}+\frac{\partial}{\partial Z}f_z(\mathbf{x})\frac{\partial Z}{\partial z} \\ &= \nabla_{\mathbf{x}}\cdot R^{-1}\cdot\mathbf{f}(\mathbf{x}).\end{aligned} \tag{S16}$$

$$\nabla f\left(R^{-1}\mathbf{r}\right) = \begin{pmatrix}\frac{\partial}{\partial x}f(\mathbf{x}) \\ \frac{\partial}{\partial y}f(\mathbf{x}) \\ \frac{\partial}{\partial z}f(\mathbf{x})\end{pmatrix} = \begin{pmatrix}\frac{\partial X}{\partial x} & \frac{\partial Y}{\partial x} & \frac{\partial Z}{\partial x} \\ \frac{\partial X}{\partial y} & \frac{\partial Y}{\partial y} & \frac{\partial Z}{\partial y} \\ \frac{\partial X}{\partial z} & \frac{\partial Y}{\partial z} & \frac{\partial Z}{\partial z}\end{pmatrix}\begin{pmatrix}\frac{\partial}{\partial X}f(\mathbf{x}) \\ \frac{\partial}{\partial Y}f(\mathbf{x}) \\ \frac{\partial}{\partial Z}f(\mathbf{x})\end{pmatrix} = R\cdot\nabla_{\mathbf{x}}f(\mathbf{x}). \tag{S17}$$

Combining Eq. (S16) and Eq. (S17) with Eq. (S15), the last expression in Eq. (S15) becomes

$$R\cdot\int_V\left[I+\frac{\nabla_{\mathbf{x}}\nabla_{\mathbf{x}}}{k^2}\right]g\left(\left|R^{-1}\mathbf{r}-\mathbf{x}'\right|\right)\cdot\mathbf{J}(\mathbf{x}')d\tau'=R\cdot\int_V\overline{G}_0\left(R^{-1}\mathbf{r},\mathbf{x}'\right)\cdot\mathbf{J}(\mathbf{x}')d\tau'. \tag{S18}$$

In the derivation of Eq. (S18), it is noticed that *I* is simply an identity operator. As a result, the commutative relation between the direct wave impedance part and the transformation operator is proved,

$$\int_V\overline{G}_0(\mathbf{r},\mathbf{r}')\cdot P_R\mathbf{J}(\mathbf{r}')dv'=P_R\int_V\overline{G}_0(\mathbf{r},\mathbf{r}')\cdot\mathbf{J}(\mathbf{r}')dv'. \tag{S19}$$

For the reflected wave part, we can express its corresponding Green's function in the Cartesian coordinate system as well as in the cylindrical coordinate system, that is,

$$\overline{G}_r^{s/p}(\mathbf{r},\mathbf{r}')=\overline{G}_r^{s/p}(\varphi,\rho,z+z'). \tag{S20}$$

In Eq. (S20), it is emphasized that the reflected wave is dependent on a relative angle φ, a transverse distance ρ and the sum of vertical distances $z+z'$ between the observation point and the source point,

$$\tan(\varphi)=\frac{y-y'}{x-x'},\ \rho=\sqrt{(x-x')^2+(y-y')^2}. \tag{S21}$$

The superscript in Eq. (S20) refers to the *s*-polarized and the *p*-polarized parts for the reflected wave. A closed form expression in the spatial domain reads,

$$\begin{aligned}\overline{G}_{r,\Box}^{s}(\mathbf{r},\mathbf{r}')&=\overline{G}_{r,\Box}^{s}(\varphi,\rho,z+z')\\&=\frac{i}{4}\begin{pmatrix}\cos2\varphi&\sin2\varphi\\\sin2\varphi&-\cos2\varphi\end{pmatrix}F_1^s(\rho,z+z')+\frac{i}{4}\begin{pmatrix}1&0\\0&1\end{pmatrix}F_2^s(\rho,z+z'),\end{aligned} \tag{S22}$$

$$\begin{aligned}\overline{G}_{r,\Box}^{p}(\mathbf{r},\mathbf{r}')&=\overline{G}_{r,\Box}^{p}(\varphi,\rho,z+z')\\&=\frac{i}{4}\begin{pmatrix}\cos2\varphi&\sin2\varphi\\\sin2\varphi&-\cos2\varphi\end{pmatrix}F_1^p(\rho,z+z')-\frac{i}{4}\begin{pmatrix}1&0\\0&1\end{pmatrix}F_2^p(\rho,z+z').\end{aligned} \tag{S23}$$

In Eq. (S22,S23), the functions $F_1^s,F_2^s,F_1^p\ and\ F_2^p$ are only dependent on the transverse and vertical distances and the detailed functional form of these functions are not of interest in this work. Note that Eq. (S22,S23) ignore the horizontal – vertical, vertical – horizontal, and vertical – vertical couplings, since they are not affected by the symmetry operations given in Eq. (S10).

Next, we apply the reflected wave Green's function to an operated current,

$$\int_V\overline{G}_r^{s/p}(\mathbf{r},\mathbf{r}')\cdot P_R\mathbf{J}(\mathbf{r}')dv'=\int_V\overline{G}_r^{s/p}(R\mathbf{x},R\mathbf{x}')\cdot R\cdot\mathbf{J}(\mathbf{x}')dv'. \tag{S24}$$

In Eq. (S24) the same change of variables as in Eq. (S15) is employed. To find a relation between the

original and transformed coordinate system, it is noticed that the rotated source coordinate **x′** and the rotated observation coordinate **x** have no effect on the vertical distance $z + z'$ and the transverse distance ρ, that is, $\rho(\boldsymbol{r}, \boldsymbol{r}') = \rho(\boldsymbol{x}, \boldsymbol{x}')$. However, the relative angle φ is altered,

$$\varphi = \theta + \varphi' \text{ for rotations,} \tag{S25}$$

$$\varphi = 2\theta - \varphi' \text{ for reflections.} \tag{S26}$$

Combining the above observations and applying to the relative angle, the transverse distance and the vertical distance, we can re-write Eq. (S22) and Eq. (S23). Take Eq. (S22) as an example,

$$\begin{aligned}\overline{G}^{s}_{r,\square}(R\mathbf{x}, R\mathbf{x}') &= \overline{G}^{s}_{r,\square}(\theta+\varphi', \rho, z+z') \\ &= \frac{i}{4}\begin{pmatrix} \cos 2(\theta+\varphi') & \sin 2(\theta+\varphi') \\ \sin 2(\theta+\varphi') & -\cos 2(\theta+\varphi') \end{pmatrix} F_1^s(\rho, z+z') + \frac{i}{4}\begin{pmatrix} 1 & 0 \\ 0 & 1 \end{pmatrix} F_2^s(\rho, z+z'),\end{aligned} \tag{S27}$$

$$\begin{aligned}\overline{G}^{s}_{r,\square}(R\mathbf{x}, R\mathbf{x}') &= \overline{G}^{s}_{r,\square}(2\theta-\varphi', \rho, z+z') \\ &= \frac{i}{4}\begin{pmatrix} \cos 2(2\theta-\varphi') & \sin 2(2\theta-\varphi') \\ \sin 2(2\theta-\varphi') & -\cos 2(2\theta-\varphi') \end{pmatrix} F_1^s(\rho, z+z') + \frac{i}{4}\begin{pmatrix} 1 & 0 \\ 0 & 1 \end{pmatrix} F_2^s(\rho, z+z').\end{aligned} \tag{S28}$$

Eq. (S27) and (S28) are respectively for rotation and reflection operations. As suggested by Eq. (S24), we right-multiply Eq. (S26) and (S27) by R. It can be proven that

$$\begin{pmatrix} \cos 2(\theta+\varphi') & \sin 2(\theta+\varphi') \\ \sin 2(\theta+\varphi') & -\cos 2(\theta+\varphi') \end{pmatrix}\begin{pmatrix} \cos\theta & -\sin\theta \\ \sin\theta & \cos\theta \end{pmatrix} = \begin{pmatrix} \cos\theta & -\sin\theta \\ \sin\theta & \cos\theta \end{pmatrix}\begin{pmatrix} \cos 2\varphi' & \sin 2\varphi' \\ \sin 2\varphi' & -\cos 2\varphi' \end{pmatrix}. \tag{S29}$$

$$\begin{pmatrix} \cos 2(2\theta-\varphi') & \sin 2(2\theta-\varphi') \\ \sin 2(2\theta-\varphi') & -\cos 2(2\theta-\varphi') \end{pmatrix}\begin{pmatrix} \cos 2\theta & \sin 2\theta \\ \sin 2\theta & -\cos 2\theta \end{pmatrix} = \begin{pmatrix} \cos 2\theta & \sin 2\theta \\ \sin 2\theta & -\cos 2\theta \end{pmatrix}\begin{pmatrix} \cos 2\varphi' & \sin 2\varphi' \\ \sin 2\varphi' & -\cos 2\varphi' \end{pmatrix}. \tag{S30}$$

Subsequently, we have

$$\overline{G}^{s}_{r,\square}(R\mathbf{x}, R\mathbf{x}') \cdot R = R \cdot \overline{G}^{s}_{r,\square}(\mathbf{x}, \mathbf{x}'). \tag{S31}$$

Similar proofs can be constructed for the *p*-polarized light as well. Substituting Eq. (S31) into Eq. (S24) immediately gives,

$$\int_V \overline{G}_r^{s/p}(\mathbf{r}, \mathbf{r}') \cdot P_R \mathbf{J}(\mathbf{r}') dv' = R \cdot \int_V \overline{G}_r^{s/p}(\mathbf{x}, \mathbf{x}') \cdot \mathbf{J}(\mathbf{x}') dv' = P_R \int_V \overline{G}_r^{s/p}(\mathbf{r}, \mathbf{r}') \cdot \mathbf{J}(\mathbf{r}') dv'. \tag{S32}$$

Hence, the commutative relation between the dyadic Green's function used in this work and the rotation and reflection symmetry operations is proved.

S3. The Rank of the Impedance Matrix and the Projected Matrices

We combine the above discussions on the eigenvalue problem with the group representation theoretical approach. The defined projection operators can be applied to all the three elements in Eq. (3, main manuscript), i.e. the impedance matrix, the excitation and the full solution. For the impedance matrix, this is equivalent to a rank decomposition where the original matrix is split into a set of rank-deficient matrices (see explanation in the supporting information). Mathematically, the original matrix is the direct sum of the projected matrices,

$$Z = \bigoplus_{j=1}^{4} Z_j,\ Z_j = P_j Z. \qquad \text{(S33)}$$

Instead of eigen-decomposing the original impedance operator as in Eq. **Error! Reference source not found.**, we can apply the eigenvalue decomposition to the projected matric Z_j. In this way, we can find the eigenmodes and eigenvalues that belong to a certain irreducible representation.

To further analyze the rank of the impedance matrix and projectd matrices, suppose that we have an impedance matrix with dimensions N by N and the eigenvalue problem for an impedance matrix is defined in Eq. (4) in the main text. Based on all the eigenmodes, we can reconstruct the impedance matrix,

$$Z = \sum_{i=1}^{N} \lambda_i \mathbf{v}_i \mathbf{v}_i^T. \qquad \text{(S34)}$$

The summation in Eq. (S34) is conducted with respect to all *N* eigenmodes. Especially, for a specific *i*, $\boldsymbol{v}_i \boldsymbol{v}_i^T$ is a matrix with a dimensions N by N but with rank one.

Since the projection operators defined in Eq. (S7) actually divide the eigenspace into subspaces, we may collect all the eigenmodes that belong to a specific projection operator, that is, a specific (row of the) irreducible representation,

$$Z = \sum_j Z_j,\ Z_j = \sum_{i=1}^{N_j} \lambda_i \mathbf{v}_i \mathbf{v}_i^T. \qquad \text{(S35)}$$

In Eq. (S35), the summation indexed by *j* is carried out over all the (rows of the) irreducible representations. For each *j*, the summation is taken up to N_j. This leads to the fact that Z_j is an N by N matrix with rank N_j. Hence, Z_j is a rank-deficient matrix.

S4. Examples

In Fig. S2 we demonstrate some exemplary eigenmodes for all three structures. For each of the structures considered in our work, we plot out of each irreducible representation one eigenmode, at a fixed chosen frequency of *f=50 THz.* It can be readily seen from this figure that the exemplary eigenmodes in the same column indeed belong to the same irreducible representation. Take the first irreducible representation (the first column in Fig. S2) as an example. Indeed, following Eq. (S6,S7) the projection operator associated with the first irreducible representation is simply the sum of all the symmetry operations,

$$P_j = \frac{1}{4}\left(P_E + P_{C_2(\pi)} + P_{m_x} + P_{m_y}\right). \quad \text{(S36)}$$

Hence, the result of this projection operator is a charge distribution which obeys all the symmetries.

For the excitation, the consequence of the projection operators is to receive the projected excitation and this excitation can only generate the current associated with the same irreducible representation,

$$P_j ZJ = P_j E \Leftrightarrow ZP_j J = P_j E \Leftrightarrow ZJ_j = E_j. \quad \text{(S37)}$$

In Eq. (S37), the second step is established because the transformation operators which are the key elements in the construction of the projection operator (as in Eq. S6) are commutative with the impedance operators. J_j and E_j are projected full solution currents and excitations due to the jth irreducible representation. Lastly, we combine the eigenmodes of the projected matrices with the projected excitation to evaluate the coupling coefficients defined in Eq. (5, main text).

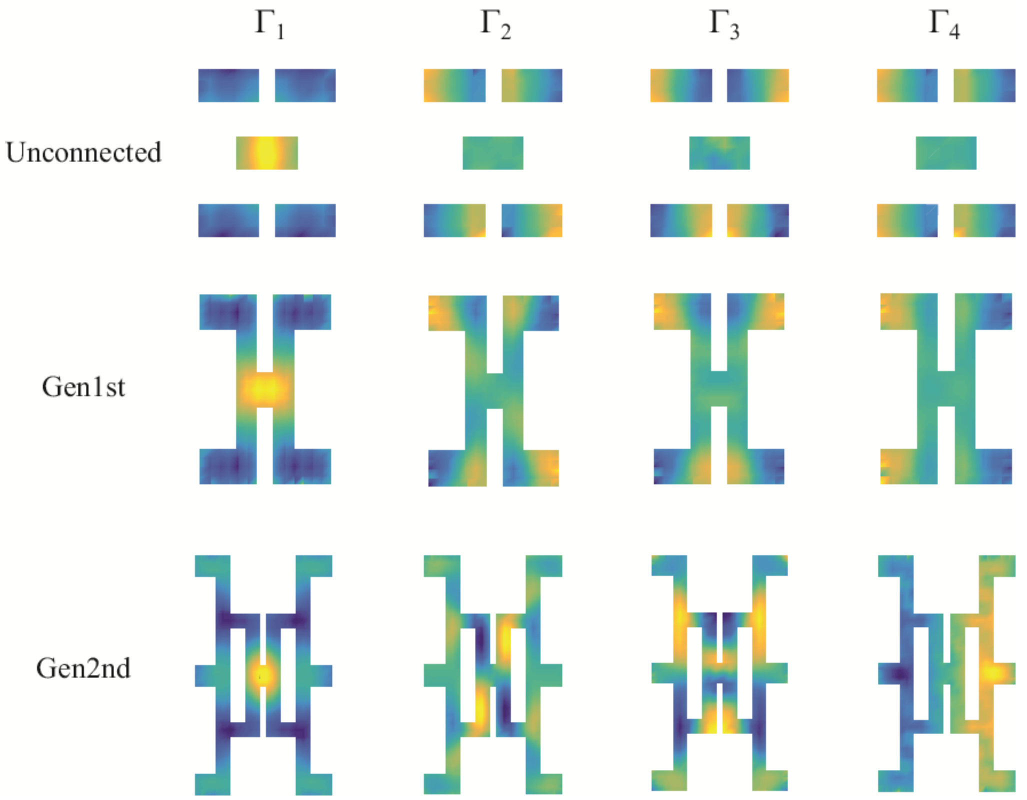

Fig. S2. Illustration of exemplary eigenmodes for the Unconnected, the 1st Generation and the 2nd Generation structures. In the columns, we find eigenmodes that belong to the same irreducible representation. In all the plots, the top surface charge is shown and coded by the blue color and the yellow color to represent the negative and positive charge accumulations. To construct this plot, we considered a fixed frequency f=50 THz, and out of each irreducible representation we plot just one of the eigenmode current distributions by way of example. To solve an actual scattering problem, one would focus on one irreducible representation commensurate with the excitation symmetry (Γ_4 in our work) and consider all the eigenvectors in that representation.

The above theoretical discussions can be immediately confirmed by numerical calculations (See

Fig. S3). The numerical calculations have been conducted by using our in-house Volumetric Method of Moments (V-MoM) tool [1-6]. For the unconnected structure, we project the excitation according to different irreducible representations (See Fig. S1(c)). It can be readily seen in Fig. S3(a) that only the projection onto the 4th irreducible representation is not zero. Accordingly, two modes, the L1 and L2 modes, associated with this irreducible representation are excited (See Fig. 3(d)). The modes belonging to the irreducible representation are not necessarily orthogonal in an inner product sense [7] and the L1 and L2 modes interfere with each other and thus generate a Fano-type line shape in the extinction spectrum in Fig. S3(a). Notice that the discontinuity in phase (Fig.S3(d)) is due to the fact that the eigenvector has one degree of freedom.

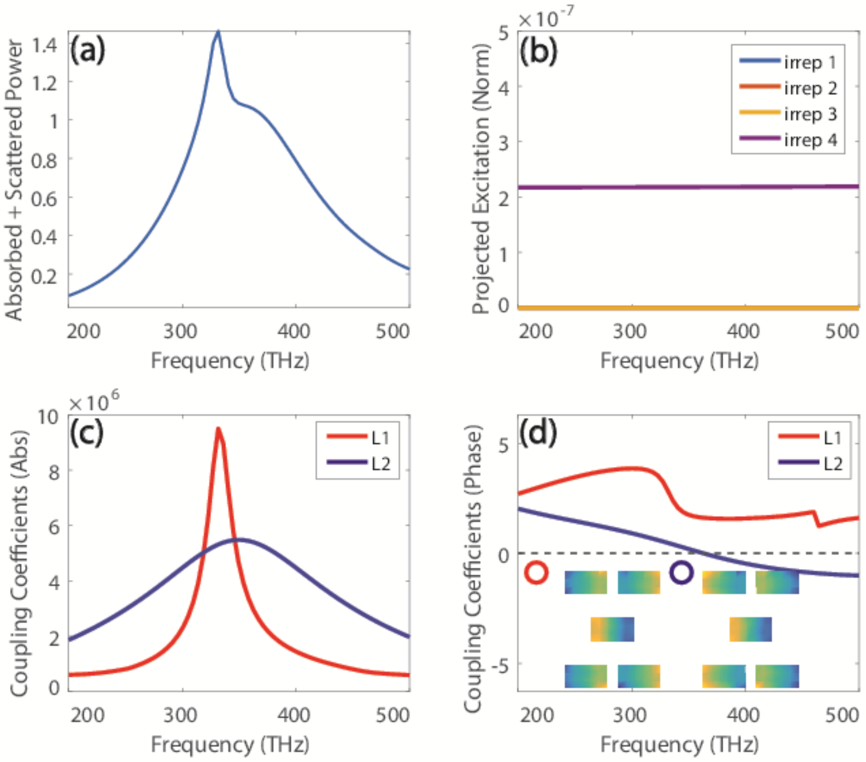

Fig. S3. Illustration of the extinction (a), the norm of the projected excitations (b), the absolute value of the coupling coefficients (c) and the phase of the coupling coefficients (d) for the Unconnected structure. In (b), the norm of the excitations projected according to different irreducible representations are marked by the blue, red, light yellow and purple color, respectively. In (c) and (d), the absolute value and the phase of the coupling coefficients for the first and second excited modes (L1 and L2) are denoted by the red and blue color respectively. In the inset of (d), the top surface charge for the L1 and L2 mode are shown and coded by the blue and yellow color to denote the negative and positive charge distribution.

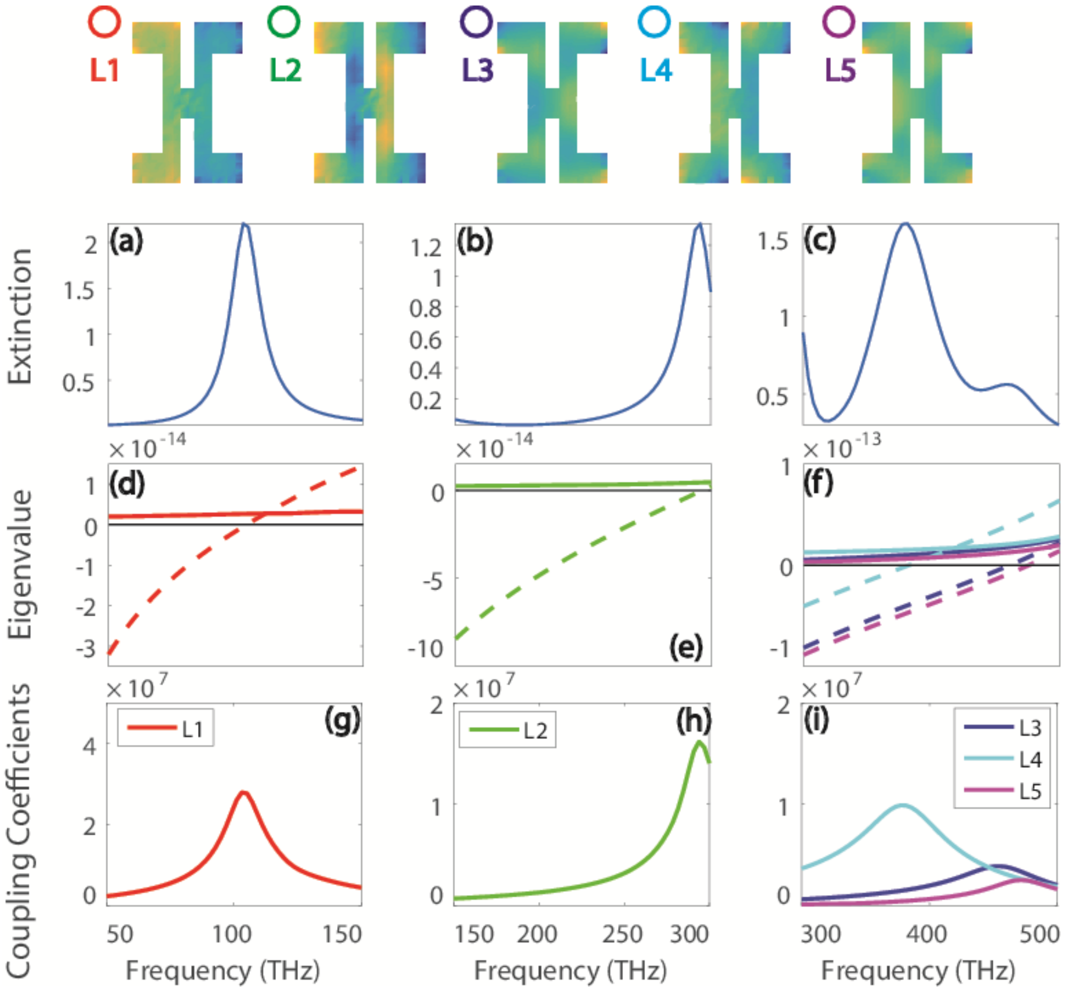

Fig. 4. Illustration of the first five eigenmodes (L1 – L5) and their corresponding eigenvalues for the 1st Generation structure. The first, second and third columns cover the frequency range: 50 THz – 150 THz, 150THz – 300 THz and 300 THz – 500 THz, respectively. (a) – (c) demonstrate the extinction power of the structure. (d) – (f) show the real (solid line) and imaginary (dashed line) parts of the eigenvalues. (g) – (i) plot the eigenmodes' coupling coefficients. Throughout the figure, the eigenmodes are marked by the red, green, blue, cyan and purple colors. Their top surface charge distributions are illustrated on the top of the figure.

A similar analysis can be carried out for the 1st Generation structure and the 2nd Generation. In Fig. S4 we demonstrate the five resonant eigenmodes (see the insets in Fig. S4 for the top surface charge distributions) for the 1st Generation structure. As suggested by Eq. (5, main text), when the absolute value of an eigenvalue becomes minimum, the corresponding eigenmode reaches its resonance. This observation is clearly reconfirmed by comparing the frequency point where the imaginary part of an eigenvalue crosses zero (see Fig. S4. (d) – (f)) yielding the resonant frequency for the coupling coefficient (see Fig. S4. (g) – (i)) and the resonant frequency for extinction (see Fig. S4. (a) – (c)).

S5. Illustration of Full Solution and Dominant Eigenmodes at Resonant Frequencies for the 2nd Generation Structure

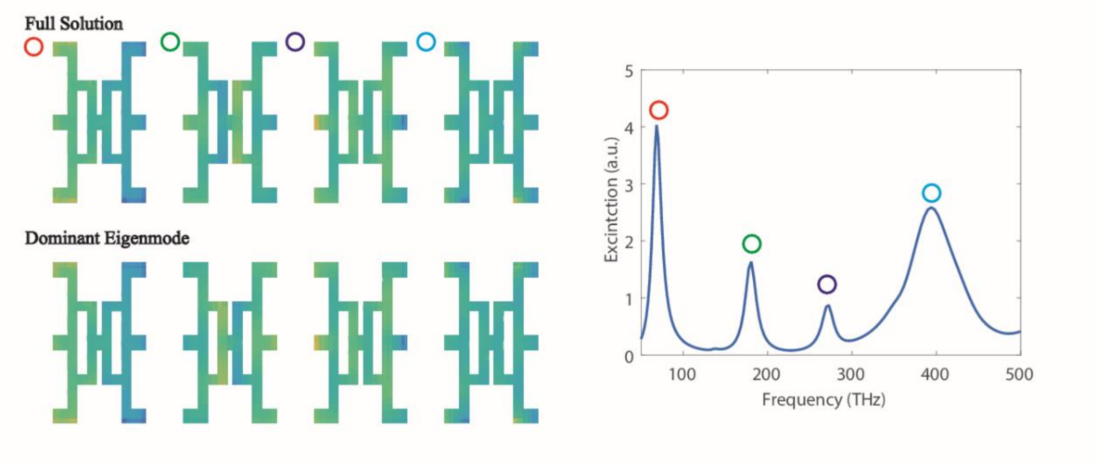

Fig. S1. Illustration of Full Solutions and Dominant Eigenmodes for the 2nd Generation Structure. The resonances are marked by the red, green, blue and cyan circles. The top surface charge distributions of the full solution and the dominant eigenmodes at these resonances are shown on the left hand side, while the extinction spectrum is shown on the right.

References:

[1] X. Zheng, V. K. Valev, N. Verellen, Y. Jeyaram, A. V. Silhanek, V. Metlushko, M. Ameloot, G. A .E. Vandenbosch, V. V. Moshchalkov, “Volumetric Method of Moments and conceptual multi-level building blocks for nano topologies,” IEEE Photonics J. **4**, 267-282 (2012).

[2] G. A. E. Vandenbosch and A. R. Van de Capelle, “Mixed-potential integral expression formulation of the electric field in a stratified dielectric medium-application to the case of a probe current source,” IEEE Trans. Antennas Propag. **40**, 806 (1992).

[3] F. J. Demuynck, G. A. E. Vandenbosch, and A. R. Van de Capelle, “The expansion wave concept–Part I: Efficient calculation of spatial Green's functions in a stratified dielectric medium,” IEEE Trans. Antennas Propag. **46**, 397 (1998).

[4] M. Vrancken, and G. A. E. Vandenbosch, “Hybrid dyadic-mixed-potential and combined spectral-space domain integral-equation analysis of quasi-3-D structures in stratified media,” IEEE Trans. Microw. Theory Techn, 55, 216-225 (2003).

[5] Y. Schols and G. A. E. Vandenbosch, “Separation of horizontal and vertical dependencies in a surface/volume integral equation approach to model quasi 3-D structures in multilayered media,” IEEE Trans. Antennas Propag. **55**, 1086 (2007).

[6] G. A. E. Vandenbosch, V. Volskiy, N. Verellen, and V. V. Moshchalkov, “On the use of the method of moments in plasmonic applications,” Radio Sci. **46**, RS0E02 (2011).

[7] X. Zheng, N. Verellen, D. Vercruysse, V. Volskiy, P. Van Dorpe, G. A. E. Vandenbosch, and V. V. Moshchalkov, “On the use of group theory in understanding the optical response of a nanoantenna,” IEEE Trans. Antennas Propag. **63**, 1589 – 1602 (2015).

[8] M. Tinkham, “Group theory and quantum mechanics,” Dover publications (2003).

[9] E. P. Wigner, “Group theory and its application to the quantum mechanics of atomic spectra,” Academic Press (1959).
